# Predicting pancreatic fistulas after pancreatoduodenectomy: development and internal validation of a new preoperative nomogram

**DOI:** 10.1016/j.clinsp.2025.100706

**Published:** 2025-06-11

**Authors:** Guilherme Naccache Namur, Fernanda Lopez Mazzucato, Ricardo Jureidini, Thiago Costa Ribeiro, Manoel de Souza Rocha, José Jukemura, Ulysses Ribeiro Junior

**Affiliations:** a Instituto do Câncer, Hospital das Clínicas da Faculdade de Medicina da Universidade de São Paulo (HCFMUSP), São Paulo, SP, Brazil; b Hospital das Clínicas da Faculdade de Medicina da Universidade de São Paulo (HCFMUSP), São Paulo, SP, Brazil

**Keywords:** Pancreatic fistula, Pancreatoduodenectomy, Risk factors, Multivariate analysis, Logistic models

## Abstract

**Introduction:**

Pancreatic Fistula (PF) is a major complication following Pancreatoduodenectomy (PD), making its prediction crucial for individualized treatment. The aim of this study is to develop a preoperative nomogram to predict PF after PD.

**Material and methods:**

From January 2009 to January 2018, patients who underwent PD were reviewed. Variables with *p* < 0.1 from univariate analysis were selected for a multivariate analysis. Variables with *p* < 0.05 were then included in the nomogram, which was internally validated in a different group of patients.

**Results:**

262 patients underwent PD during the period of the study. The risk factors for PF identified in the derivation group were male gender (OR = 2.89), higher BMI (OR = 1.14) and pancreatic duct diameter ≤ 3 mm (OR = 3.52). Preoperative weight loss exceeding 10 % within six months was protective against PF (OR = 0.16). A preoperative nomogram incorporating these four variables was developed and demonstrated good performance in the validation set, with an area under the ROC curve of 0.798.

**Conclusions:**

male gender, a main pancreatic duct ≤ 3 mm and higher BMI are risk factors for PF, while weight loss is protective. The developed nomogram accurately predicts PF occurrence using these variables.

## Highlights

• A preoperative nomogram predicts Pancreatic Fistula (PF) after pancreatoduodenectomy.
• Risk factors for PF: male gender, BMI, and pancreatic duct ≤ 3 mm.
• Weight loss > 10 % is protective against PF development.
• The nomogram shows strong predictive accuracy (AUC = 0.798).

## Introduction

Despite a relevant reduction in mortality after Pancreatoduodenectomy (PD) during the last four decades,^1^ morbidity after this procedure is still high, usually ranging from 30 % to 50 %, and most complications are related to Pancreatic Fistula (PF).^2^ The introduction of the International Study Group on Pancreatic Fistula (ISGPF) definition was a crucial step in standardizing research in this area,^3^ as PF incidence could vary between 10 % and 29 % within the same cohort depending on the diagnostic criteria used.^4^ In 2016, the ISGPF updated its consensus, reclassifying what was previously known as grade A PF to biochemical leak.^5^

Some risk factors have been associated with PF, including pancreatic duct diameter,^6^, ^7^, ^8^ pancreatic parenchyma texture[6,7–10] and excess weight.^10^, ^11^, ^12^ However, many of these studies were conducted prior to the ISGPF consensus update and often included what is now classified as a biochemical leak.^13^ Callery Et al. developed the Fistula Risk Score (FRS) which incorporates definitive pathology and three intraoperative variables: pancreatic duct width, gland texture, and blood loss.^6^^,^^13^ Despite its good performance after external validation,^14^ the FRS is not suitable for preoperative prediction of PF, although some authors have correlated radiological exams with pancreatic fibrosis or steatosis,^15^^,^^16^ to overcome this inadequacy. Other authors have developed preoperative scores to predict PF after PD, however, some of these scores failed external validation or are dependent on very complex mathematical calculations.^7^^,^^17^

A preoperative risk score for PF, which could be easily calculated, could significantly aid patients in the consenting process for such complex procedures. It would enable surgeons to better manage the postoperative course and hospital stay, as well as to more effectively select candidates for future interventions aimed at preventing PF.

Therefore, the aim of this study is to develop a preoperative risk score and nomogram to predict the occurrence of PF after pancreatoduodenectomy and to perform internal validation of this tool.

## Material and methods

This study was performed according to strict ethical principles of the Declaration of Helsinki. It was approved by the ethics committee (Comissão de Ética para Análise de Projetos de Pesquisa do Hospital das Clínicas da Faculdade de Medicina da Universidade de São Paulo ‒ CAPPesq) under the on-line protocol number 13,015 and the approval number was 940.582. Informed consent was waived by the ethics committee (CAPPesq) which understands that the consent given by patients authorizing the inclusion of clinical data in a local database is sufficient for observational studies.

A prospective maintained database was retrospectively reviewed for patients who underwent PD at the Instituto do Câncer do Hospital das Clínicas da Faculdade de Medicina da Universidade de São Paulo. The study design adhered to the Transparent Reporting of a Multivariate Prediction Model for Individual Prediction or Diagnosis (TRIPOD) guidelines.^18^

A total of 266 consecutive patients who underwent PD from February 2009 to February 2018 were included in the analysis. These patients were divided into two groups based on the date of their procedure. The risk factors analysis was conducted using data from patients operated on between 2009 and 2016 (derivation group). The nomogram was then developed and internally validated in a different group of patients who underwent PD between 2016 and 2018 (validation group). A sample size of at least 80 patients was considered sufficient for testing the nomogram.^19^ Patients who underwent additional pancreatic resection, such as distal pancreatectomy or enucleation, simultaneously with PD were excluded from the study.

The preoperative variables included in the study were gender, age, clinical diagnosis, preoperative biliary drainage, smoking and alcohol consumption, ASA classification, ECOG performance score, Body Mass Index (BMI), preoperative history of weight loss within six months preceding surgery, bilirubin levels, hemoglobin levels, and serum albumin levels. Intraoperative variables assessed were operative time, blood transfusion requirements, blood loss, total infused volume, hemodynamic instability, and tissue perfusion biomarkers, such as venous lactate and central venous oxygen saturation.

Two experienced radiologists reviewed Computed Tomography (CT) scans and/or Magnetic Resonance Imaging (MRI) for the study. Measurements included pancreatic duct diameter (in millimeters) and pancreatic neck thickness (in centimeters) with their ratio calculated. Additionally, the anteroposterior and latero-lateral diameters of the abdomen at the level of the umbilical scar were measured in centimeters and the ratio between these dimensions was calculated. In CT scans, the attenuation of the pancreatic parenchyma in the body of the pancreas was assessed during the non-enhanced phase. This was done using the largest possible circular area, excluding the main pancreatic duct, to evaluate gland texture.

Pancreatic reconstruction was performed using a separate jejunal loop for the anastomosis. The technique for the anastomosis itself was described by Shrikhande et al.^20^ At least one peritoneal drain was inserted, and patients did not receive somatostatin analogs during the perioperative period.

The primary outcome was the occurrence of PF, defined according to the 2016 ISGPF consensus.^5^ PF was characterized by the drainage of fluid with an amylase concentration three times the upper limit of normal, through a drain placed during or after surgery, starting after the third postoperative day. The diagnosis required significant alterations in the postoperative course, such as the use of antibiotics, percutaneous drainage, reoperations, or the need for drain placement longer than three weeks.

### Statistics

Categorical variables were presented as frequencies, and their association with PF was assessed using chi-square tests or Fisher's exact tests. Quantitative variables were reported as means or medians, and their association with PF was evaluated with Student's *t*-test or Mann-Whitney tests. The bivariate Odds Ratio (OR) with a 95 % Confidence Interval (95 % CI) for each variable was estimated using simple logistic regression.^21^ Variables with data loss greater than 20 % were not considered for multivariate analysis. A backward stepwise regression was applied with 5 % input and output threshold criteria to select the final model to explain the occurrence of PF. Statistical analysis was performed using the SPSS program (SPSS Inc., Chicago IL, USA). Independent risk factors identified were used to develop a risk score, which was graphically represented by a nomogram (Stata®). The performance of the nomogram in predicting PF was evaluated in the validation group using a ROC curve analysis (95 % CI).

## Results

A total of 266 patients underwent PD from 2009 to 2018. Four patients were excluded from the study due to other additional pancreatic resections. The remaining 180 patients were assigned to the derivation group, and 82 patients were assigned to the validation group. The mean age was 59.2 years in the derivation group and 59.8 years in the validation group. The mean BMI was 25.5 kg/m^2^ in the derivation group and 25.1 kg/m^2^ in the validation group. In both groups, the most common diagnosis was ampullary carcinoma. Epidemiological data are presented in Table 1. Pancreatic Fistula (PF) occurred in 19.4 % of patients in the derivation group and 25.5 % of patients in the validation group.

**Table 1:** Characteristics of derivation and validation sets.

	Derivation Group (*n* = 180)	Validation Group (*n* = 82)
**Age, years, ±SD**	59.2 ± 13.9	59.8 ± 12.7
**Gender, male, n ( %)**	79 (43.9)	40 (48.8)
**BMI, Kg/m^2^, ±SD**	25.49 ± 4.5	25.10 ± 5.4
**Weight loss > 10 %, n ( %)**	63 (35)	40 (49)
**Pancreatic duct diameter ≤3mm, n ( %)**	88 (51.5)	47 (57.3)
**Diagnosis, n ( %)**		
Pancreatic Adenocarcinoma	39 (21.6)	13 (15.8)
Chronic Pancreatitis	3 (1.9)	1 (1.2)
Ampullary carcinoma	67 (37.2)	34 (41.5)
Cholangiocarcinoma	11 (6.1)	8 (9.8)
Neuroendocrine Tumor	23 (12.7)	7 (8.5)
Duodenal Carcinoma	11 (6.1)	4 (4.9)
Cystic Neoplasia	26 (14.4)	15 (18.3)
**Pancreatic Fistula, n ( %)**	35 (19.4)	21 (25.5)
Grade B	31 (17.2)	20 (24.3)
Grade C	4 (2.2)	1 (1.2)

SD, Standard Deviation; BMI, Body Mass Index.

Table 2 presents the results of the univariate analysis for demographic, laboratory, and intraoperative data, while Table 3 summarizes the results of univariate analysis for radiological data. PF was associated with younger age, male gender, higher BMI, diagnosis other than pancreatic cancer, prolonged surgical time, pancreatic duct diameter ≤ 3 mm, thicker pancreas, and higher attenuation of pancreatic parenchyma in unenhanced CT scan. Preoperative weight loss greater than 10 % within six months prior to surgery was identified as a protective factor against PF. These variables were included in the multivariate analysis, which revealed that a pancreatic duct ≤ 3 mm (OR = 3.52, 95 % CI 1.34–9.26), male gender (OR = 2.89, 95 % CI 1.16–7.18) and higher BMI (OR = 1.14, 95 % CI 1.03–1.26) were independent risk factors for PF. Conversely, preoperative weight loss greater than 10 % (OR = 0.16, 95 % CI 0.05–0.56) was identified as a protective factor (Table 4).

**Table 2:** Univariate analysis of demographic, laboratory and intraoperative data.

	Pancreatic Fistula		Total	OR	95 % CI		p
	No	Yes			Inferior	Superior	
**Age, years**	**(*n*=145)**	**(*n*=35)**	**(*n*=180)**	0.97	0.95	1.00	**0.018**^b^
mean ± SD	60.5 ± 12.7	54.3 ± 17.2	59.2 ± 13.9				
**Gender, n ( %)**							**0.078**
Female	86 (59.3)	15 (42.9)	101 (56.1)	1.00			
Male	59 (40.7)	20 (57.1)	79 (43.9)	1.94	0.92	4.10	
**BMI (Kg/m^2^)**	**(*n*=142)**	**(*n*=35)**	**(*n*=177)**	1.07	0.99	1.16	**0.079**^b^
mean ± SD	25.2 ± 4.3	26.7 ± 5.1	25.5 ± 4.5				
**Diagnosis, n ( %)**							**0.018**
Pancreatic adenocarcinoma and Chronic Pancreatitis	40 (27.6)	3 (8.6)	43 (23.9)	1.00			
Other diagnosis	105 (72.4)	32 (91.4)	137 (76.1)	4.06	1.18	14.02	
**Weight loss ≥10 %, n ( %)**							**0.001**
No	84 (58.7)	30 (88.2)	114 (64.4)	1.00			
Yes	59 (41.3)	4 (11.8)	63 (35.6)	0.19	0.06	0.57	
**Alcoholism, n ( %)**							0.466^a^
No	134 (92.4)	34 (97.1)	168 (93.3)	1.00			
Yes	11 (7.6)	1 (2.9)	12 (6.7)	0.36	0.05	2.87	
**Smoking, n ( %)**							0.981
No	108 (74.5)	26 (74.3)	134 (74.4)	1.00			
Yes	37 (25.5)	9 (25.7)	46 (25.6)	1.01	0.43	2.35	
**Biliary drainage, n ( %)**							0.297
No	93 (65)	26 (74.3)	119 (66.9)	1.00			
Yes	50 (35)	9 (25.7)	59 (33.1)	0.64	0.28	1.48	
**ASA, n ( %)**							0.423
1 e 2	102 (70.3)	27 (77.1)	129 (71.7)	1.00			
3 e 4	43 (29.7)	8 (22.9)	51 (28.3)	0.70	0.30	1.67	
**ECOG, n ( %)**							0.576^a^
0 e 1	126 (86.9)	32 (91.4)	158 (87.8)	1.00			
2 e 3	19 (13.1)	3 (8.6)	22 (12.2)	0.62	0.17	2.23	
**Bilirubin, mg/dL**	**(*n*=143)**	**(*n*=32)**	**(*n*=175)**	0.95	0.88	1.04	0.191^c^
mean ± SD	3.5 ± 6.3	2.2 ± 4.8	3.3 ± 6				
**Positive bile culture, n ( %)**							0.510
No	37 (37.8)	5 (29.4)	42 (36.5)	1.00			
Yes	61 (62.2)	12 (70.6)	73 (63.5)	1.46	0.48	4.46	
**Hemoglobin, g/ dL**	**(*n*=145)**	**(*n*=35)**	**(*n*=180)**	1.18	0.97	1.45	0.101^b^
mean ± SD	12.1 ± 1.9	12.7 ± 2.1	12.2 ± 2				
**Albumin, g/dL**	**(*n*=103)**	**(*n*=26)**	**(*n*=129)**	1.18	0.75	1.86	0.472^b^
mean ± SD	4 ± 0.8	4.2 ± 1	4.1 ± 0.9				
**Operative time, minutes**	**(*n*=145)**	**(*n*=35)**	**(*n*=180)**	1.01	1.00	1.01	**0.009**^b^
mean ± DP	321.6 ± 76.6	363.1 ± 109.6	329.6 ± 85.3				
**Blood transfusion, n ( %)**							0.274
No	117 (80.7)	31 (88.6)	148 (82.2)	1.00			
Yes	28 (19.3)	4 (11.4)	32 (17.8)	0.54	0.18	1.65	
**Vascular Resection, n ( %)**							0.532^a^
No	129 (89)	33 (94.3)	162 (90)	1.00			
Yes	16 (11)	2 (5.7)	18 (10)	0.49	0.11	2.23	
**Blood Loss, mL**	**(*n*=66)**	**(*n*=15)**	**(*n*=81)**	1.000	0.999	1.002	0.594^c^
mean ± DP	587.1 ± 370.4	650 ± 369.8	598.8 ± 368.8				
**Hemodynamic instability, n ( %)**							0.895
No	123 (84.8)	30 (85.7)	153 (85)	1.00			
Yes	22 (15.2)	5 (14.3)	27 (15)	0.93	0.33	2.66	

Chi-square test;.

a Fisher's exact test;.

b *t*-Student test;.

c Mann-Whitney test.

**Table 3:** Univariate analysis of radiological data.

	Pancreatic Fistula		Total	OR	95 % CI		p
	No	Yes			Inferior	Superior	
**Pancreatic duct diameter, n ( %)**							**0.002**
≤ 3 mm	62 (45.6)	26 (74.3)	88 (51.5)	3.45	1.50	7.91	
> 3 mm	74 (54.4)	9 (25.7)	83 (48.5)	1.00			
**Pancreatic thickness, cm**	**(*n*=136)**	**(*n*=35)**	**(*n*=171)**	2.01	1.03	3.92	**0.039**^a^
mean ± SD	1.9 ± 0.6	2.1 ± 0.5	1.9 ± 0.6				
**Duct diameter/Pancreatic thickness ratio**	**(*n*=136)**	**(*n*=35)**	**(*n*=171)**	0.06	0.01	0.59	**0.007**^a^
mean ± SD	0.28 ± 0.22	0.18 ± 0.19	0.26 ± 0.22				
**Unenhanced CT pancreatic attenuation, Hu**	**(*n*=122)**	**(*n*=32)**	**(*n*=154)**	1.04	1.00	1.08	**0.071**^a^
mean ± SD	32.2 ± 16.3	37.7 ± 9.9	33.3 ± 15.3				
**AP diameter at umbilicus, cm**	**(*n*=136)**	**(*n*=35)**	**(*n*=171)**	1.00	0.91	1.11	0.946^a^
mean ± SD	23.1 ± 3.6	23.2 ± 3.7	23.1 ± 3.6				
**LL diameter at umbilicus, cm**	**(*n*=136)**	**(*n*=35)**	**(*n*=171)**	1.02	0.92	1.14	0.715^a^
mean ± SD	31.7 ± 3.5	32 ± 3.4	31.8 ± 3.4				
**AP/LL ratio**	**(*n*=136)**	**(*n*=35)**	**(*n*=171)**	0.45	0.00	46.24	0.740^a^
mean ± SD	0.73 ± 0.08	0.72 ± 0.08	0.73 ± 0.08				

Chi-square test;.

a *t*-Student test. CT, Computed Tomography; Hu, Hounsfield units; AP, Anteroposterior; LL, Laterolateral.

**Table 4:** Multivariate analysis result.

	OR	95 % CI		p
		Inferior	Superior	
**Gender (male)**	2.89	1.16	7.18	**0.022**
**BMI (Kg/m^2^)**	1.14	1.03	1.26	**0.016**
**Weight loss ≥10 %, n ( %)**	0.16	0.05	0.56	**0.004**
**Pancreatic duct diameter, n ( %)**	3.52	1.34	9.26	**0.011**

Stepwise backward logistic regression.

The risk score derived from the multivariate analysis can be calculated using the following formula:

$$\begin{array}{c} PFprobability\\ =exp[-3.504+1.249*\left(malegender\right)--1.1933*\left(weightloss\right)--1.851\\ {}*\left(ductdiameter\left(mm\right)\right)+0.105*\left(BMI\left(Kg/m2\right)\right)]/[1+exp\left(...\right)] \end{array}$$

**Fig. 1:**
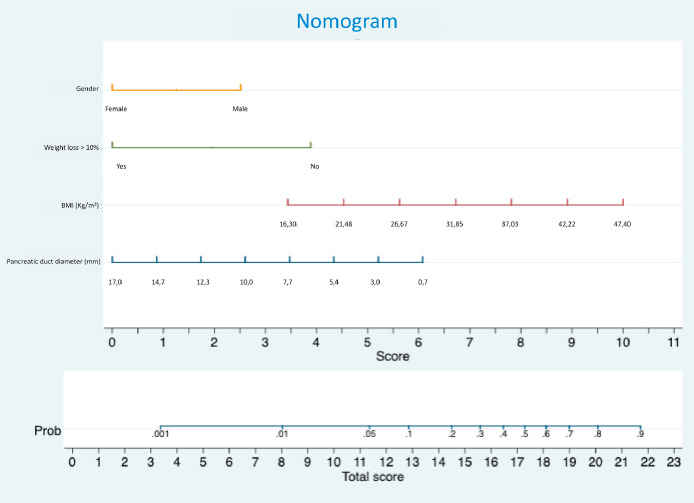
Nomogram to predict PF after PD.

**Fig. 2:**
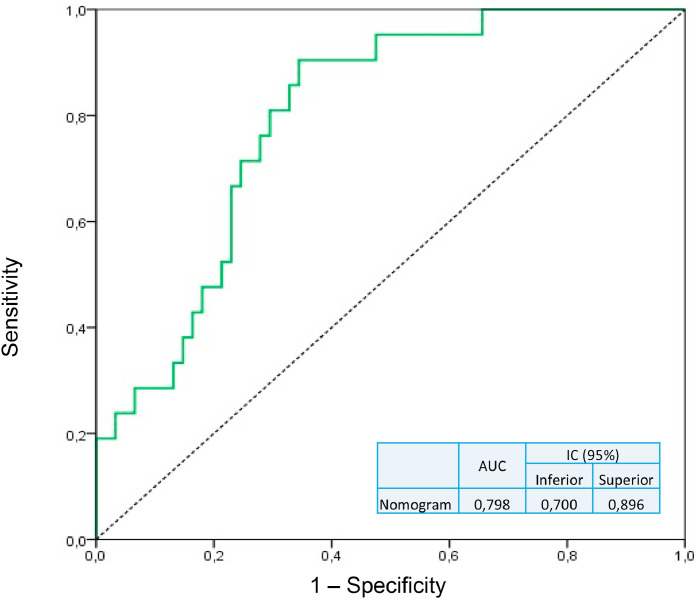
ROC of risk score on validation set.

The nomogram (Fig. 1) incorporates independent variables identified in the multivariate analysis and provides a graphical representation of the logistic regression model. Internal validation was performed by applying the nomogram in the validation set of patients. The area under the receiver operator curve was 0.798, with a 95 % Confidence interval between 0.700 and 0.896 (Fig. 2)

## Discussion

Predicting postoperative Pancreatic Fistula (PF) is critically important, as it represents the most significant complication following Pancreaticoduodenectomy (PD),^2^ leading to increased hospital costs^22^ and negatively affecting oncological outcomes.^23^ Numerous strategies, including the use of somatostatin analogs and external stents, have been investigated to reduce the incidence of PF after PD.^24^, ^25^, ^26^ However, the quality of evidence supporting these interventions remains low. An important limitation in many studies is the lack of patient stratification by PF risk or the reliance on a single risk factor for stratification. Consequently, these trials may have included a disproportionate number of low-risk patients, potentially introducing bias into the findings.

The authors assessed risk factors associated with the development of postoperative Pancreatic Fistula (PF) following Pancreaticoduodenectomy (PD) and identified male gender, higher Body Mass Index (BMI), and a pancreatic duct diameter ≤ 3 mm as independent risk factors through multivariate analysis. Conversely, preoperative weight loss exceeding 10 % of total body weight within 6-months prior to surgery was a protective factor against PF. Based on these findings, the authors developed and validated a preoperative nomogram to predict the likelihood of PF after PD. This tool may enhance patient consent by providing a clearer risk assessment and guiding surgical decision-making throughout the perioperative period. High-risk patients, as identified by the nomogram, may benefit from interventions such as external stents,^27^ whereas low-risk patients could be considered for early drain removal,^28^ or even the omission of drains altogether.^29^ Moreover, this nomogram may assist surgeons in predicting PF after minimally invasive PD, as intraoperative evaluation of pancreatic texture is not feasible. Additionally, this nomogram could prove useful in selecting patients for future trials aimed at reducing the incidence of postoperative PF.

This is not the first study to develop a risk score for predicting pancreatic fistula.^6^, ^7^, ^8^, ^9^^,^^12^^,^^17^^,^^30^ Yamamoto et al.^17^ proposed an additive preoperative risk score ranging from 0 to 7, based on five variables: two clinical (gender and diagnosis of pancreatic cancer) and three radiological (duct diameter relative to parenchymal thickness, portal vein involvement, and the anteroposterior diameter of the abdominal cavity). However, additive scores can’t calculate the probability of fistula, but rather only help in identifying low and high-risk patients. The performance of this score was not adequate after external validation, with an area under the curve of 0.61 with a 95 % CI of 0.42 to 0.80.^14^

Roberts et al.^7^ developed a logistic score incorporating pancreatic duct width and BMI to estimate the likelihood of postoperative PF, which performed well during external validation.^14^ However, the present study identified an additional risk factor, male sex, and a protective factor, preoperative weight loss, which were integrated into a graphical nomogram. This tool offers a more comprehensive and intuitive understanding of preoperative risk, enhancing risk stratification and patient management.

Callery et al.^6^ created the Fistula Risk Score (FRS), one of the most extensively studied scores for predicting postoperative Pancreatic Fistula (PF). It incorporates four variables: duct width, pancreatic texture, intraoperative blood loss, and pathology, classifying patients into four distinct risk categories. Although the FRS demonstrated strong performance in external validation,^14^ blood loss is more indicative of intraoperative difficulties rather than an independent risk factor for PF.

Although the primary objective of this study was to develop a preoperative nomogram, intraoperative variables such as surgical time, bleeding, vascular resection, and hemodynamic instability were included in the risk analysis. Only prolonged surgical time was associated with the occurrence of PF in the univariate analysis, though this association did not remain significant in the multivariate analysis. Preoperative variables were included because studies have shown that bleeding can be an independent risk factor for pancreatic fistula.^6^ Furthermore, it was crucial to assess whether any of these variables had a substantial impact on the occurrence of fistula, as their omission could significantly reduce the nomogram's efficiency.

Several studies have identified an association between a smaller diameter of the main pancreatic duct and the occurrence of postoperative Pancreatic Fistula (PF), whether assessed intraoperatively after transection of the pancreatic neck,^6^ or preoperatively through radiological exams.^7^^,^^8^^,^^11^ Risk scores have utilized duct diameter in different ways: some as a categorical variable[6,30] and others as a continuous variable.^7^^,^^7^^,^^15^^,^^31^ It is hypothesized that thinner pancreatic ducts are risk factors for PF because they are related to less parenchymal fibrosis, making pancreatojejunostomies technically more challenging.^9^ In the present study, the diameter of the main pancreatic duct was measured from preoperative radiological exams and was included as a dichotomous variable in the multivariate analysis, thereby enhancing the model's performance.^19^ The analysis revealed that a diameter of ≤3 mm was an independent risk factor for PF. Consequently, it was incorporated into the nomogram as a continuous variable, improving the score’s accuracy and providing a better graphical representation of its impact on overall risk.

This is not the first study to demonstrate that a higher BMI is associated with an increased risk of postoperative PF.^10^, ^11^, ^12^ In this study, BMI was included in the nomogram as a continuous variable, thereby providing a clearer representation of its impact on the risk of PF.

Due to mechanisms that are not totally understood, the male gender is associated with increased surgical morbidity in various abdominal procedures, including pancreatectomies and colectomies.^12^^,^^17^^,^^30^ This association may be attributed to differences in fat distribution, which is often more visceral in men.^32^ Attempts to estimate visceral fat by measuring the anteroposterior and laterolateral abdominal diameters and calculating their ratios did not reveal an association with a higher incidence of postoperative PF. Tranchart et al.^33^ associated a higher area of visceral fat with PF, however, their method was laborious, as it involved drawing the entire visceral fat area manually and required specific software.

While weight loss is generally recognized as a risk factor for postoperative complications after PD,^34^ the present study found that weight loss was protective against PF This finding aligns with reports from Wellner et al. and Ellis et al.^9^^,^^11^ Weight loss is common in patients with pancreatic cancer^35^ often resulting from malabsorption due to pancreatic exocrine insufficiency,^36^ which is highly prevalent among these patients and tends to worse over time^37^ due to loss of pancreatic parenchyma.^38^ The observed lower incidence of PF among patients with weight loss greater than 10 % within six months prior to surgery may be attributable to this progressive parenchymal loss.

Nomograms are valuable tools for performing complex calculations without the need for computers, making them convenient for use by surgeons in an office setting. In addition, because they are the graphical representation of a risk score, they enable a better understanding of the importance of a given variable on the overall risk. You et al. developed a nomogram to predict the occurrence of PF using only preoperative variables, however, the nomogram performance, after internal validation, could be considered somehow unsatisfactory, and 95 % CI was not reported.^39^

Internal validation of the nomogram developed in this study demonstrated good diagnostic performance in predicting the occurrence of PF, with an Area Under the Curve (AUC) of 0.798 (95 % CI 0.7–0.896). Diagnostic tests with AUC greater than 50 % have some discriminatory capacity; those with areas greater than 70 % are acceptable; and those exceeding 80 % have excellent diagnostic performance.^40^

This study has some limitations. Firstly, this is a retrospective study, and therefore, associations observed should not be interpreted as causation. Secondly, the selection of patients for the derivation and validation group was based on the date of the procedure. However, since surgical technique and postoperative care remained constant throughout the study period, there was minimal risk of bias. Lastly, although this nomogram performed well in internal validation, it lacks external validation. External validation is important to confirm the generalizability and robustness of predictive models, as they often demonstrate reduced performance when applied to external cohorts. However, such validation was beyond the scope of the present study and should be addressed in future research.

## Conclusions

In conclusion, based on four preoperative variables, pancreatic duct diameter, BMI, male gender and over 10 % weight loss, it was possible to develop a preoperative nomogram for PF with adequate reliability and accuracy.

## Funding

This research did not receive any specific grant from funding agencies in the public, commercial, or not-for-profit sectors.

## CRediT authorship contribution statement

**Guilherme Naccache Namur:** Conceptualization, Methodology, Data curation, Writing – original draft, Project administration. **Fernanda Lopez Mazzucato:** Investigation, Data curation. **Ricardo Jureidini:** Conceptualization. **Thiago Costa Ribeiro:** Investigation, Data curation. **Manoel de Souza Rocha:** Conceptualization, Investigation. **José Jukemura:** Methodology, Writing – review & editing, Supervision. **Ulysses Ribeiro Junior:** Conceptualization, Methodology, Writing – review & editing, Supervision.

## Declaration of competing interest

The authors declare no conflicts of interest.
